# Ecological Drivers of Vertebrate Richness and Implications for Inland Wetland Survey in Korea

**DOI:** 10.3390/ani16030419

**Published:** 2026-01-29

**Authors:** Yein Lee, Minkyung Kim, Jae Geun Kim, Sangdon Lee

**Affiliations:** 1 Department of Environmental Science & Engineering, Ewha Womans University, Seoul 03760, Republic of Korea; hanna.lee3@gmail.com (Y.L.); enviecol@ewha.ac.kr (M.K.); 2 Department of Biology Education, Seoul National University, Seoul 08826, Republic of Korea; jaegkim@snu.ac.kr; 3 Center for Education Research, Seoul National University, Seoul 08826, Republic of Korea

**Keywords:** inland wetland, amphibians, reptiles, birds, mammals, species richness, generalized linear model (GLM), generalized additive model (GAM), wetland dataset, South Korea

## Abstract

Wetlands have been recognized as nature-based solutions to the climate crisis. This study evaluates the state of standardization in nationwide inland wetland survey datasets and analyzes terrestrial vertebrate patterns by integrating datasets with public environmental data. Species richness data for amphibians/reptiles (432 wetlands), birds (1183 wetlands), and mammals (72 wetlands) were compiled from 134 reports published between 2000 and 2021. Using generalized linear models (GLMs) and generalized additive models (GAMs), we assessed how 15 explanatory variables (climate, topography, wetland information, land use, and water quality) relate to species richness. Model families were chosen for each taxonomic group, and variables were selected using the Akaike information criterion (AIC) and ecological plausibility. Deviance explained was 55.5% for amphibians/reptiles, 60.1% for birds, and 52.4% for mammals. Wetland area and Normalized Difference Vegetation Index (NDVI) were positively associated with species richness across all groups. Despite the large volume of survey data, inconsistent reporting formats and limited metadata constrain longitudinal and time series analyses. Standardized protocols and metadata management are therefore needed to build a systematic national database that can support wetland ecological modeling and conservation policy.

## Simple Summary

The Republic of Korea has conducted inland wetland surveys for more than 20 years. However, much of the information is available mainly in report documents, which makes cross-site comparison and long-term analysis difficult. We compiled 134 survey reports published between 2000 and 2021, calculated species richness for amphibians/reptiles, birds, and mammals in each wetland, and tested how richness relates to climate, topography, land use, and water quality. Species richness was generally higher in larger wetlands and where vegetation was healthier, and these patterns remained after accounting for spatial structure. Yet the report-based data is not sufficiently standardized, limiting reuse and the ability to track change over time. These results support the need for standardized survey records and an integrated, analysis-ready national wetland database.

## 1. Introduction

Wetlands are areas that are flooded or saturated with water for extended periods, keeping the soil moist and supporting vegetation adapted to these conditions [1,2,3]. The productivity of wetlands rivals that of tropical rainforests and coral reefs [4,5], providing essential water and habitats for numerous plant and animal species [6]. In addition, wetlands are recognized as indispensable ecosystems in the context of the climate crisis because they deliver a broad range of ecosystem services, including carbon storage, water purification, flood mitigation, freshwater provision, and groundwater recharge [6,7]. Accordingly, under the Ramsar Convention, the international community emphasized and protected the ecological and social values of wetlands. In line with these global trends, the Republic of Korea also enacted the Wetlands Conservation Act in 1999 and has conducted statutory wetland surveys since 2000.

Nature-based solutions that leverage nature’s capacity to address and mitigate climate crises have recently gained significant attention. Wetland ecosystems are recognized as crucial to this approach [8,9,10]. However, in Korea, recognition of the importance of wetlands for biodiversity conservation and ecosystem services has increased in recent years, yet national-scale studies integrate wetland biodiversity patterns with environmental drivers remain limited [10,11]. In particular, non-standardized survey datasets have constrained robust cross-site comparisons and long-term synthesis.

Wetlands are complex ecosystems in which climate, soil, and hydrological conditions are tightly interconnected [12,13]. Inland wetlands occurring along lakes and rivers, in mountainous areas, and on lowland floodplains function as ecotones between terrestrial and freshwater environments [2]. Owing to their shallow water depth, high soil moisture, and proximity to groundwater, their hydrological and water-quality conditions respond to changes in surrounding land use and precipitation. Wetland-associated terrestrial vertebrate species richness is generally shaped by habitat size and heterogeneity, landscape structure, and climatic constraints. Therefore, at the national scale, standardized, analysis-ready datasets that enable quantitative assessment of key ecological drivers and biotic variation across wetlands are essential, as they provide an evidence base for conservation priorities. Particularly at the policy stage, prioritizing wetland restoration and conservation or assessing ecosystem service values requires a standardized national-level database [6,14,15,16]. Such a database can serve as a core foundation linking long-term monitoring, research, and management.

This study reviews the status and potential applications of national-level wetland data and quantifies key ecological drivers of terrestrial vertebrate species richness focusing on statutory inland wetland survey datasets in Korea. First, we examine the scope and organization of inland wetland survey data compiled at the national level, including their structure and schema. Second, this study examines whether the established data are sufficiently standardized and quantified to be practically employed in applied and statistical studies, identifying problems uncovered during the analysis process. Third, for the quantitative analysis, this work applies generalized linear models (GLMs) and generalized additive models (GAMs) by integrating inland wetland biota data with public environmental data (e.g., climate, topography, and soil) to assess the effects of external environmental factors on patterns of terrestrial vertebrate taxa. Because observations may be spatially structured at the national scale, we test for spatial autocorrelation in model residuals and, where needed, use spatially explicit models to avoid inflated inference. Finally, based on structural limitations identified through this analysis, we propose standardization and technical improvements to support the future development of a nationally integrated wetland database.

## 2. Materials and Methods

### 2.1. Study Area

The study area comprises the Republic of Korea, in the southern part of the Korean Peninsula in East Asia (33°06′ to 38°45′ N, 124°11′ to 131°52′ E). The total peninsular area is 100,364 km^2^ [17], of which 70% comprises mountainous terrain [17]. The Republic of Korea has a temperate monsoon climate. During winter, this area is influenced by continental cold and dry air masses, whereas in summer, it is affected by maritime warm and humid air masses. The annual average temperature ranges from about 10 °C to 15 °C, and the annual average precipitation is between 1000 and 1800 mm [18].

Korea has been conducting statutory surveys by classifying wetlands as inland or coastal wetlands (tidal flats) [19]. According to the Wetlands Conservation Act of Korea, inland wetlands include freshwater wetlands such as lakes, marshes/swamps, rivers, and river estuaries (river mouths), whereas coastal wetlands refer to intertidal flats between the high and low-tide water marks [2,19]. This study focuses on the analysis of inland wetlands. The analysis covers all inland wetlands in South Korea (2704 sites, 1153.4 km^2^), including riverine (1326 sites, 990.7 km^2^), lacustrine (635 sites, 99.0 km^2^), mountainous (466 sites, 13.8 km^2^), and artificial (277 sites, 49.9 km^2^) wetlands [2].

### 2.2. National Inland Wetland Survey Data: Compilation and Standardization Assessment

We reviewed the statutory National Inland Wetland Survey (NIWS) to understand the current status and characteristics of the data structure. Inland wetlands are conserved and managed under the Wetland Conservation Act (1999) [20]. Although several agencies undertake site or jurisdiction-based monitoring, the National Institute of Ecology (NIE) Wetland Center provides the only continuous, nationwide survey dataset with a consistent reporting framework [2], making it the primary source for cross-site comparisons and standardization assessment in this study.

To understand the NIWS framework (survey components, scope, and cycle), we analyzed the Inland Wetland Survey Guidelines (2021) [21]. We then evaluated the availability, accessibility, and usability of the dataset accumulated through the NIWS. We manually digitized wetland-level biotic records from 134 NIWS reports and compiled them into an Excel database. To assess data availability and the level of standardization, we examined the datasets available for download from EcoBank (as of October 2025) and compared whether key metadata present in the original reports were retained in the downloadable datasets. The assessment covered both data availability (e.g., bulk-download availability and linkage to wetland history/records) and the presence of key metadata required for analysis.

### 2.3. Response Variables (Species Richness)

Species richness (SR), defined as the number of unique species recorded per wetland (presence-based), regardless of individual counts, was used as the response variable in this study. This study targets terrestrial vertebrates, including amphibians/reptiles, birds, and mammals, and independent analyses were conducted for each taxonomic group. Wetlands are a transitional zone between terrestrial and aquatic systems [9,22] and are characterized by complex interactions between the climate, soil, and hydrological factors. Terrestrial vertebrates were selected as the subject taxon for this study because they move between wetlands and the surrounding areas, participating in reproduction, predation, and material cycling [23,24]. These vertebrates are comprehensively affected by environmental conditions, including wetland landscape, hydrology and water quality, habitat connectivity, and surrounding land use.

Amphibians/reptiles primarily inhabit wetlands. Due to their low mobility, they are sensitive to changes in wetland water quality, depth, and temperature, and they can indirectly reflect the health of the wetland micro-environment [23]. In this study, amphibians and reptiles were grouped into a single category for analysis based on the raw data classification. Birds respond sensitively to structural complexity, vegetation cover, open water area, and hydrological connectivity [25]. In addition, birds use diverse habitats and travel long distances, and the wetland size and surrounding landscape influence their distribution. Finally, mammals create diverse microhabitats and increase spatial heterogeneity through various activities, such as foraging, predation, and burrowing, leading to cascading changes in associated biological communities [26]. In addition, because mammals mediate energy flow and primarily correspond to higher trophic levels in food chains [26,27], they can serve as indirect indicators of habitat health.

Species data for taxonomic groups were extracted and employed based on the results of biota surveys documented in NIWS reports (2000 to 2021, N = 134). Portable document format (PDF) reports were compiled into an Excel database, and data preprocessing focused on correcting errors and excluding ambiguous records. The taxonomic groups (e.g., bird data included in the mammal taxonomic data) were separated and removed. Species names were standardized based on the National Species List of Korea from the National Institute of Biological Resources to eliminate confusion due to synonyms and historical names. In addition, wetland data reconstructed into the Excel database were removed if they did not match the current wetland names or contained missing values.

Wetland surveys select target wetlands according to annual and regional plans. Therefore, although some wetlands may be resurveyed for history tracking and re-evaluation, the same wetlands are not repeatedly surveyed every year (The number of surveyed wetlands included in this study is summarized in Appendix A Table A1 by year and taxonomic group). Accordingly, this study was designed as a nationwide cross-sectional assessment rather than a time-series analysis. To avoid disproportionate influence from a small number of repeatedly surveyed wetlands, species richness was summarized at the wetland level by averaging year-specific richness values to derive mean species richness (SR_mean). Final sample sizes were 432 wetlands for amphibians/reptiles, 1183 wetlands for birds, and 72 wetlands for mammals.

### 2.4. Environmental Explanatory Variables

The explanatory variables in the statistical analysis were selected by reviewing the Inland Wetland Survey Guidelines (2021) [21] from the NIE and previous literature. We compiled survey items potentially associated with SR from the Guidelines and previous literature by taxonomic groups and retained only variables that could be constructed from publicly available national data. Data sources and access links for each variable are provided in Table 1 and the corresponding references.

The explanatory variables were categorized into five types: wetland information, topography, land use, climate, and water environment. In total, 15 environmental variables were selected (Table 1). Wetland information represents the area and type of each wetland, whereas topographic factors (digital elevation model [DEM], aspect, and Topographic Wetness Index [TWI]) describe the wetland topography and hydrological characteristics. Land-use/land-cover variables (urban [built-up/developed area], agricultural, and forest cover and the Normalized Difference Vegetation Index [NDVI]) characterize the landscape structure around wetlands. NDVI was obtained from the Landsat-based NDVI product (30 m) distributed by the Korea Institute of Geoscience and Mineral Resources (KIGAM) for 2019. NDVI was summarized using zonal statistics, calculating the mean value within each wetland boundary polygon. Because wetland polygons often include open-water surfaces (NDVI near zero or negative), polygon-mean NDVI can be lower than values typically reported for terrestrial vegetation. Climate factors, including annual temperature, precipitation, relative humidity, and wind speed, describe habitat conditions. Water environment factors (biochemical oxygen demand [BOD] and water temperature) reflect water-quality characteristics. Environmental predictors were compiled from the best-available national layers, each representing a specific reference period (e.g., a representative year, climate normals, or multi-year averages); the source period, aggregation, and the rationale for using the corresponding reference period for each layer are provided in Appendix A Table A2). These layers were used to characterize typical site conditions for cross-sectional inference, and we avoided interpreting the fitted models as year-wise changes in SR.

All variables were constructed in ArcGIS Pro [28]. Table 1 summarizes the units, ranges, and sources of each variable. For continuous variables, the final variables were selected after reviewing multicollinearity (VIF < 5). Wetland area was log-transformed to mitigate scale effect.

**Table 1:** Environmental explanatory variables in generalized linear model analysis.

Category	Variables	Unit	Range	Source
Wetland information	Wetland area (log)	m^2^	4.16–17.93	Wetland inventory 2704 [29]
	Wetland type	Four types (riverine, artificial, mountainous, lacustrine)		Wetland inventory 2704 [29]
Topographic	Altitude	m	0–1670	SRTM DEM 30 m [30]
	Aspect	Unitless index	−1.00–1.00	SRTM DEM 30 m [30]
	TWI	Unitless index	5.19–26.07	SRTM DEM 30 m [30]
Land use/ land cover	Urban cover	%	0–75.6	Land-cover map [31]
	Agricultural cover	%	0–95.41	Land-cover map [31]
	Forest cover	%	0–100	Land-cover map [31]
	NDVI	Unitless index	−0.30–0.50	NDVI/LANDSAT 8 [32]
Climate	Annual temperature	°C	6.97–15.87	Bioclim_BIO1 [33]
	Annual precipitation	mm	1060–2170	Bioclim_BIO12 [33]
	Relative humidity	%	59.65–79.57	Climatological normals 91′–20′ [34]
	Average wind speed	m/s	1.18–6.69	Climatological normals 91′–20′ [34]
Water environment	BOD	mg/L	0–7.14	Water-quality monitoring 16′–23′ [35]
	Water temperature	°C	0.02–21.21	Water-quality monitoring 16′–23′ [35]

Notes: BOD: biochemical oxygen demand, DEM: digital elevation model, NDVI: Normalized Difference Vegetation Index, TWI: Topographic Wetness Index. Wetland area was log-transformed prior to modeling.

### 2.5. Statistical Analysis

This study first explored bivariate relationships using scatterplots with ordinary least squares trend lines for visualization only, to identify candidate environmental associations of terrestrial vertebrate SR in wetlands. This work applied GLMs and GAMs with SR as the response variable to estimate the effects of the explanatory variables. Due to differences in ecological characteristics and the data distribution of taxonomic groups, the models were constructed independently for each group (amphibians/reptiles, birds and mammals).

The general form of the GLMs is as follows:

$$g\left(\mu_{i}\right)=\alpha +\sum_{k=1}^{p}\beta_{k}X_{ik}$$

**Figure A1:**
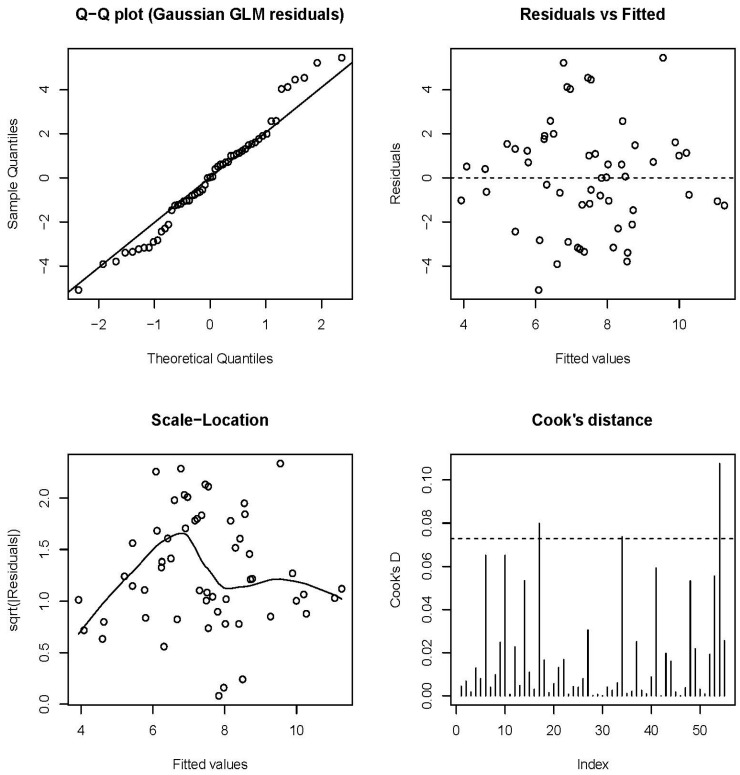
Diagnostic plots for the final Gaussian generalized linear model (Gaussian GLM) of mammal mean species richness (SR_mean; N = 72 wetlands). Panels show a Q–Q plot of residuals, residuals versus fitted values, a scale–location plot (√|residuals| vs. fitted values), and Cook’s distance for assessing influential observations. Overall, no strong deviations from normality, constant variance, or influential outliers were detected.

In this equation, *g*(∙) denotes the link function, *u_i_* is the expected SR for wetland *i, a* is the intercept, *X_ik_* is the *k*th environmental variable (explanatory variable) for wetland *i*, and *β_k_* is the corresponding regression coefficient. During model fitting, overdispersion was evident for amphibians/reptiles (N = 432) and birds (N = 1183); therefore, we used negative binomial models with a log link. For mammals (N = 72), we fitted a Gaussian GLM with an identity link because mammal SR showed continuous variation with little evidence of overdispersion unlike the amphibian/reptile and bird data. Model assumptions were evaluated using standard residual and influence diagnostics (Appendix A Figure A1). Across all wetlands with mammal SR data, zero values were rare; therefore, zero-inflated count models were not pursued. As a sensitivity check, we compared the Gaussian model with count-family alternatives fitted to an integer version of the response (Appendix A Table A3).

Spatial autocorrelation was assessed using Moran’s I on model residuals with a k-nearest neighbor spatial weights matrix (k = 8, row-standardized) and Monte Carlo permutation tests (1000 simulations). Sensitivity to neighborhood size was evaluated using k = 6 and 10, yielding consistent inference (Appendix A Table A4). Residual Moran’s I indicated significant positive spatial autocorrelation for the bird and amphibian/reptile negative binomial GLMs (NB-GLMs). Therefore, we refitted these two models as negative binomial generalized additive models (NB-GAMs) by adding a two-dimensional smooth of geographic coordinates, specified as s(Xm, Ym, k = 80) with smoothing parameters estimated by restricted maximum likelihood (REML) to capture broad-scale spatial structure. Moran’s I was recalculated on GAM residuals to confirm that spatial autocorrelation was mitigated. For mammals, residual Moran’s I was not significant; therefore, the Gaussian GLM was retained without an added spatial smooth.

All models considered the 15 explanatory variables summarized in Table 1. Stepwise model selection based on Akaike information criterion (AIC) was used to derive a parsimonious final model. Model fit was assessed using AIC and residual deviance, and explanatory power was quantified as explained deviance:

$$Explained\;deviance\left(\%\right)=1-\frac{Residual\;deviance}{Null\;deviance}\times 100.$$

For each taxonomic group, we reported statistical significance of predictors in the final selected model at *p* < 0.05. All statistical analyses were performed in R v4.5.1 [36].

## 3. Results

### 3.1. Data Availability and Standardization Gaps in the Inland Wetland Dataset

This analysis evaluated the provision status, accessibility, usability, and standardization of datasets accumulated through NIWS. The findings reported below are based on datasets that could be retrieved as of October 2025.

The final output of the NIWS is the Inland Wetland Survey Reports published since 2000. A total of 154 volumes were produced between 2000 and 2024. These reports contain comprehensive wetland survey information, including wetland status, biota survey results covering more than 11 taxonomic groups, topography, sediment characteristics, and water-quality measurements (Appendix A Table A5). They also provide field-specific in-depth investigations, detailed raw field data, and evaluations and recommendations for conservation management. However, this information is provided in document format, limiting its direct reuse for quantitative analyses that require standardized analysis ready tables [37,38,39].

Data compiled through the NIWS are provided through EcoBank, operated by the NIE (nie-ecobank.kr). Reports from 2019 to 2024 are directly accessible via EcoBank, whereas reports compiled from 2000 to 2018 are distributed across public archives. Table 2 summarizes the formats and attributes of contents currently available. In practice, EcoBank mainly provides polygon spatial layers for inland wetlands (.shp) and associated map-based services and selected outputs such as species lists (.xlsx), vegetation map (.shp), and report files (.pdf).

Critically, EcoBank-downloadable tabular biota datasets represent only a subset of the report-based survey outputs (Table 2) and are heavily focused on species occurrence/abundance records. While the original NIWS reports include core metadata (e.g., survey dates, methods, site information, and survey frequency) that would support reconstruction of temporal structure and effort-aware interpretation, the downloadable datasets typically retain only a limited set of attributes. This simplification results in the loss of analysis-critical metadata such as survey dates, transect/segment identifiers, occurrence locations, survey frequency, and sampling effort. Consequently, time series cannot be reconstructed (e.g., by year, season, or month), and effort correction is not possible, raising concerns about bias in relative comparisons of abundance or occurrence across wetlands. Overall, these gaps constrain the usability of the current NIWS-derived datasets for a broad range of quantitative and applied research.

### 3.2. Baseline Patterns of Species Richness Across Inland Wetlands

**Figure 1:**
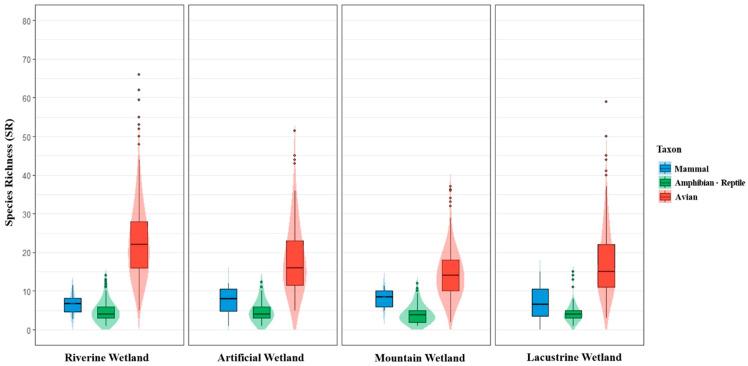
Species richness (SR) by wetland type and taxon as combined violin and boxplots. Panels represent riverine, artificial, mountain, and lacustrine wetlands; colors distinguish mammals (blue), amphibians/reptiles (green), and birds (red). The y-axis is SR. Boxes reveal the median and interquartile range (IQR) with whiskers at 1.5 × IQR; violin plots indicate the density of values. Dots represent individual wetland observations, allowing comparisons of central tendency, spread, and skewness across wetland types and taxa.

**Figure 2:**
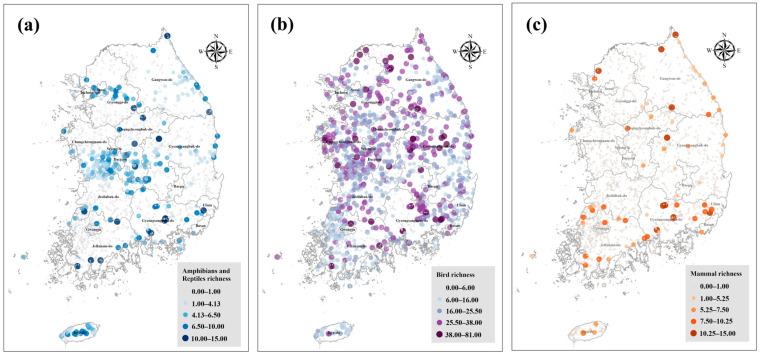
Map of the distribution of species richness of (**a**) amphibians/reptiles, (**b**) birds, and (**c**) mammals. Each dot represents one surveyed wetland. Color denotes the richness class at that site. Classes are divided into five bins per taxon based on observed values (see legends). Administrative boundaries are presented for reference. Color breaks differ by panel.

Based on data from the NIWS established to date, we conducted a baseline analysis to describe the fundamental status and SR patterns of inland wetlands. We analyzed 134 reports published from 2000 to 2021 and calculated the SR as the number of species recorded per wetland. The range and distributional shape of the wetland SR by taxonomic group were examined using boxplots and violin plots (Figure 1). Figure 2 maps wetland SR onto the national wetland layer (N = 2704 sites).

For terrestrial vertebrates, SR was calculated as the number of species surveyed in each wetland. The number of wetlands included for each taxonomic group varied depending on data availability and preprocessing results. SR was calculated for 432 wetlands for, 1183 for birds, and 72 for mammals.

Amphibian/reptile SR ranged from 1 to 15 species (Mean = 4.5 (±2.7)) and was predominantly concentrated in riverine and lacustrine wetlands in major river systems, such as the Han, Geum, and Nakdong Rivers (Figure 2a). Among wetlands in the top 10% of amphibian/reptile SR (SR ≥ 8, N = 48), riverine wetlands accounted for 54% (N = 26), mountain wetlands for 20.8% (N = 10), artificial wetlands for 14.6% (N = 7), and lacustrine wetlands for 10.4% (N = 5; Figure 1). Furthermore, wetlands with top-10% amphibian/reptile SR were predominantly lowland sites at average elevations of 0 to 700 m, except for a single mountain wetland on Jeju (Sumunmulbangdui Wetland; elevation 1006 m). This pattern was concentrated at lower elevations relative to the overall elevation range of all wetlands (0 to 1670 m).

Among terrestrial vertebrate taxa, birds exhibited the widest SR range, from 2 to 81 species (Mean = 19.22 (±9.3)). Spatially, bird SR was widely distributed nationwide (Figure 2b). Among wetlands in the top 10% of bird SR (SR ≥ 31, N = 118), 70.3% (N = 83) were riverine wetlands (Figure 1), with high concentrations along the Nakdong Rivers and Western Coastal estuaries. Other wetland types followed in the order of lacustrine wetlands (13.6%, N = 16), artificial wetlands (10.2%, N = 12), and mountain wetlands (5.9%, N = 7). The highest SR within each wetland type was recorded at Cheonsu Bay Estuary Wetland (riverine; SR = 81), Mungyeong Doline Wetland (lacustrine; SR = 59), Sangju Gonggeomji Wetland (artificial; SR = 51.5), and Jeongeup Wolyeong Wetland (mountain; SR = 37). Each of these wetlands exhibited the highest SR within its respective wetland type.

Finally, mammal SR ranged from 0 to 15 species (Mean = 7.1 (±3.0)). Mammal richness was predominantly high in wetlands in northern Gangwon Province, eastern Gyeongsangbuk-do and Gyeongsangnam-do, and southern Jeollanam-do. Conversely, low SR values were observed in the Seoul metropolitan area and central inland regions (Figure 2c). Among wetlands with 10 or more recorded species (N = 15), mountain wetlands accounted for 40.0% (N = 6), riverine wetlands for 26.7% (N = 4), lacustrine wetlands for 20.0% (N = 3), and artificial wetlands for 13.3% (N = 2). The wetland with the highest mammal SR was the Mungyeong Doline Wetland (SR = 15).

### 3.3. Ecological Drivers of Species Richness (GLM/GAM Results)

This section presents the ecological drivers of species richness and implications derived from the data structure and its limitations. Because residual spatial autocorrelation was detected for amphibians/reptiles and birds in the NB-GLMs, we present NB-GAMs as the final models for these groups. After adding spatial smooth, residual Moran’s I was no longer significant, indicating that spatial dependence was effectively mitigated (Appendix A Table A4). For mammals, residual Moran’s I from the Gaussian GLM was not significant. Therefore, we retained the Gaussian GLM with an identity link without an added spatial smooth. Overall model performance (explained deviance) was 55.5% for amphibians/reptiles, 60.1% for birds, and 52.4% for mammals (Table 3). Model parameter estimates for each taxonomic group are reported in Table 4.

#### 3.3.1. Amphibians and Reptiles

**Figure 3:**
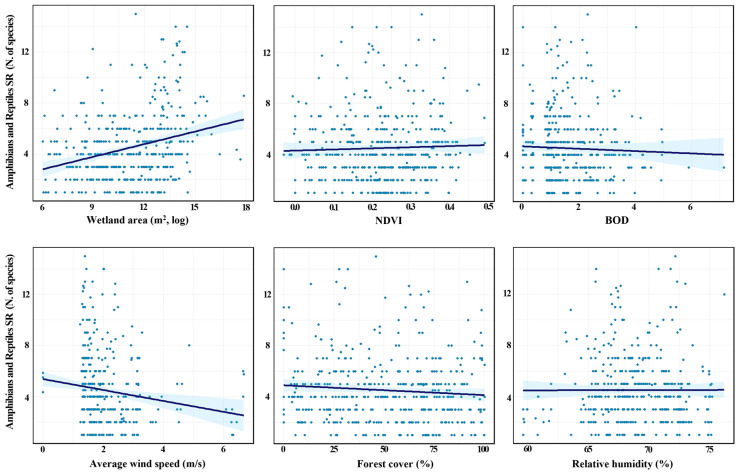
Relationships between amphibian/reptile species richness (SR; number of species) and explanatory variables (wetland area (m^2^, log), Normalized Difference Vegetation Index (NDVI), biochemical oxygen demand (BOD), forest cover (%), average wind speed (m/s), and relative humidity (%)) based on a simple linear regression. Each plot represents the bivariate relationship between SR and a single explanatory variable without controlling for other factors.

We analyzed the relationship between the SR of amphibians/reptiles and key environmental variables. We visualized bivariate associations using scatterplots with simple linear regression lines for selected predictors (Figure 3). In these bivariate plots, when examining simple linear regression, wetland area (m^2^, log) indicated a strong positive association, whereas the NDVI displayed a weak positive association. By contrast, BOD, forest cover (%), and average wind speed (m/s) showed negative bivariate associations with SR (Figure 3).

Amphibians/reptiles SR was modeled as a function of environmental predictors using an NB-GAM with a log link, including a two-dimensional smooth of coordinates s(Xm,Ym) to account for spatial structure (Table 4). In the final model, SR increased with wetland area (log-transformed; *β* = 0.0742, *p* < 0.001) and NDVI (*β* = 0.7355, *p* = 0.0198). After accounting for spatial structure, the remaining covariates were not statistically significant (*p* > 0.05). The spatial smooth term was highly significant (edf = 37.33, *p* < 0.001), and the model explained 55.5% of deviance (adjusted R^2^ = 0.516; Table 3). Residual spatial autocorrelation was no longer significant after including the spatial smooth (Appendix A Table A4).

#### 3.3.2. Birds

**Figure 4:**
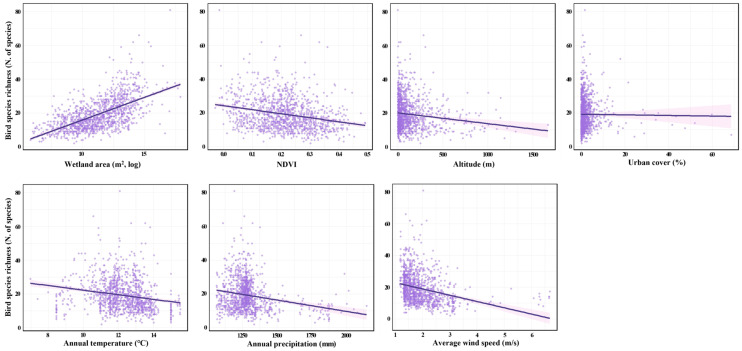
Relationships between bird species richness (SR; number of species) and explanatory variables (wetland area (m^2^, log), Normalized Difference Vegetation Index (NDVI), altitude (m), urban cover (%), annual temperature (°C), annual precipitation (mm), and average wind speed (m/s)) based on a simple linear regression. Each plot represents the bivariate relationship between SR and a single explanatory variable without controlling for other factors.

The relationships between bird richness and critical environmental variables were analyzed. To visualize bivariate patterns, we produced scatterplots of SR against individual environmental variables with simple linear regression lines (Figure 4). When examining simple linear regression, the wetland area (m^2^, log) showed a strong positive association with bird richness, whereas NDVI, altitude (m), annual temperature (°C), annual precipitation (mm), and average wind speed (m/s) showed negative associations. Urban cover (%) revealed a weak negative association (Figure 4).

Birds SR was modeled using an NB-GAM with a log link, including a two-dimensional smooth of wetland coordinates s(Xm, Ym) to account for spatial structure (Table 4). In the final model, bird SR showed a strong positive association with wetland area (log-transformed; β = +0.1353, *p* < 0.001) and a positive association with NDVI (β = +0.3187, *p* = 0.025) (Table 4). Notably, NDVI showed a negative bivariate trend but became positive after accounting for spatial structure and other covariates. Urban cover showed a weak negative trend but did not reach statistical significance at α = 0.05 (β = −0.0041, *p* = 0.068), and the remaining covariates were not significant after accounting for spatial structure (*p* > 0.05). The spatial smooth term was highly significant (edf = 46.29, *p* < 0.001), and the model explained 60.1% deviance (adjusted R^2^ = 0.558; Table 3). Residual spatial autocorrelation was not significant after including the spatial smooth (Appendix A Table A4).

#### 3.3.3. Mammals

**Figure 5:**
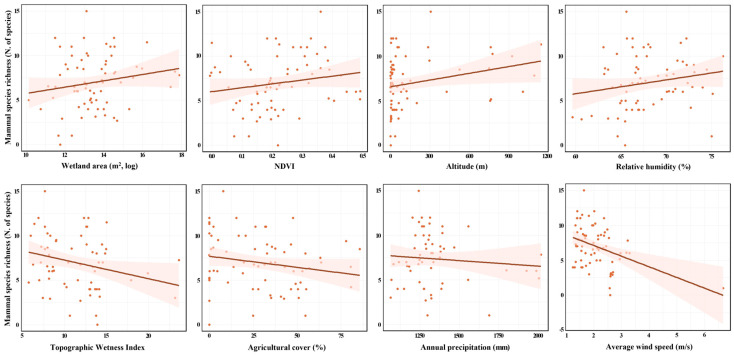
Relationships between mammal species richness (SR; number of species) and explanatory variables (wetland area (m^2^, log), Normalized Difference Vegetation Index (NDVI), altitude (m), relative humidity (%), Topographic Wetness Index (TWI), agricultural cover (%), annual precipitation (mm), and average wind speed (m/s) based on a simple linear regression. Each plot represents the bivariate relationship between SR and a single explanatory variable without controlling for other factors.

In the final Gaussian GLM for mammals, wetland area (m^2^, log), NDVI, altitude (m), relative humidity (%), TWI, agricultural cover (%), annual precipitation (mm), and average wind speed (m/s) were significant predictors of mammal SR. Scatterplots with simple linear regression lines were used to visualize their bivariate relationships with SR (Figure 5). When examining simple linear regression, wetland area (m^2^, log), NDVI, altitude (m), and relative humidity (%) showed positive associations, whereas TWI, agricultural cover (%), annual precipitation (mm), and average wind speed (m/s) showed negative associations (Figure 5).

Controlling for other variables (Table 4), mammal SR increased significantly with higher NDVI (*β* = 9.52, *p* < 0.05). Wetland area and relative humidity also had positive effects (wetland area: *β* = 0.71, *p* < 0.05, relative humidity: *β* = 0.31, *p* < 0.05), and altitude (m) showed a weak, but significant positive effect (*β* = 0.007, *p* < 0.05).

In contrast, mammal SR decreased with increasing average wind speed and TWI (average wind speed: *β* = −2.57, *p* < 0.05; TWI: *β* = −0.28, *p* < 0.05). Mammal SR was also negatively associated with agriculture cover (%) and annual precipitation (mm) (agricultural cover: *β* = −0.06, *p* < 0.05; annual precipitation: *β* = −0.008, *p* < 0.05).

## 4. Discussion

### 4.1. Model Performance Comparison

This study presents an analysis of SR status by taxonomic group (amphibians/reptiles, birds, and mammals) for each wetland in Korea by aggregating the number of species recorded in survey reports (134 volumes total) published from 2000 to 2021.

In taxon-specific final models, explained deviance was 55.5% for amphibians/reptiles, 60.1% for birds, and 52.4% for mammals (Table 3). Residuals spatial autocorrelation was assessed using Moran’s I on Pearson residuals. Significant spatial autocorrelation was detected for amphibians/reptiles and birds in the initial negative binomial GLMs, but it was mitigated after adding a two-dimensional spatial smooth term in the negative binomial GAMs, whereas mammals showed no residual spatial autocorrelation.

For amphibians/reptiles, a model analysis of terrestrial vertebrates across Spain [40] presented similar explanatory power: 23.4% for amphibians and 29.4% for reptiles. However, local-scale studies that explicitly incorporate breeding sites, fine-scale wetland microhabitat conditions (e.g., water-level fluctuations and hydroperiods), water-quality parameters, and the presence/absence can achieve higher explanatory power (30–50%) [41,42]; therefore, our results should be interpreted with due regard to the limitations of nationwide, averaged predictors. For birds, the explained deviance (60.1%) falls within the range reported in national and regional GLM studies (30–60%) [40,43], while studies incorporating nonlinearities/interactions or machine-learning approaches sometimes exceed 60% [44,45]. For mammals, explained deviance was 52.4%, but interpretation warrants caution given the small sample size (N = 72); previous large-scale studies have reported values around 30% [40].

### 4.2. Drivers of Amphibian/Reptile Richness

The SR of amphibians/reptiles is primarily concentrated in river estuaries (river mouths) of major river basins and lowland wetlands (0 to 700 m) near lakes. In the final NB-GAM, SR increased with wetland area and NDVI (Table 4). The two-dimensional spatial smooth was also significant, indicating broad-scale geographic structure not captured by the measured predictors. Although bivariate plots suggested associations with water-quality and climatic variables, these effects were not retained as significant after accounting for covariates and spatial structure.

The positive area effect is consistent with higher richness in lager wetlands and in wetlands associated with large water bodies [46]. In addition, stabilized water levels and residence time are thought to enhance spawning and larval survival, particularly in amphibians [47]. NDVI as proxy for vegetation greenness, likely reflects food and shelter availability through vegetation and invertebrate supported resources [48], though responses may vary among reptile taxa [49].

Water-quality and land-cover variables showed limited support in the final model, but this does not preclude ecological importance. Rather, these relationships may require finer-resolution hydrological and water-quality indicators and habitat descriptors, together with improved effort-aware survey metadata, to better resolve taxon-specific drivers in future analyses.

### 4.3. Drivers of Birds Richness

Bird SR across Korea’s inland wetlands had the widest distribution range among terrestrial vertebrates, with high richness sites (top 10%) concentrated in riverine wetlands (70.3%). This pattern highlights the importance of river estuaries (river mouths) and floodplains as productive, and their function as habitats and resting sites for birds. This pattern likely reflects the strong environmental gradients and habitat mosaics in river mouths and floodplain settings, where freshwater interacts with brackish influences can create a diverse foraging and resting conditions [50]. This finding aligns with prior studies indicating that river mouths and floodplains provide heterogeneous habitat conditions and function as important stopover habitats for migratory birds [50,51].

Regarding environmental determinants, the wetland area (log) showed a consistent, positive effect on bird SR, supporting the typical species–area relationship. Larger wetlands are more likely to include greater environmental and habitat heterogeneity (e.g., larger wetland surface area, variation in vegetation cover, the presence and extent of open-water areas and more complex landscape composition and landscape heterogeneity), which can increase the number of available ecological niches and facilitate species coexistence [51,52,53,54]. In this context, NDVI can be interpreted as indicators related to vegetation that may contribute to habitat diversity.

In the final negative binomial GAM, bird species richness showed positively associated with wetland area and NDVI. The spatial smooth term was significant, and residual spatial autocorrelation was mitigated after including the spatial term (Moran’s I on GAM residuals was not significant). Urban cover and climate variables had only weak effects in the final model, and average wind speed, previously suggested as an important constraint, was not significant after accounting for spatial structure and covariates.

### 4.4. Drivers of Mammal Richness

Mammal SR was generally low in metropolitan and central inland regions, whereas higher values were more often observed in mountainous and artificial wetlands. In the final Gaussian GLM, SR increased with wetland area, NDVI, relative humidity, and altitude and decreased with average wind speed, agricultural cover, TWI, and annual precipitation. Large wetlands with greener vegetation may provide greater access to food, shelter, and seasonal resources [55,56], whereas higher agricultural cover can reduce habitat area, increase habitat fragmentation, and introduce anthropogenic disturbances, potentially limiting mammal persistence [57,58,59]. Higher wind speeds can inhibit mammal activity by making animals harder to detect and, ecologically, by disrupting scent trails in crucial foraging areas, increasing heat loss, and raising energy use [60,61].

Moreover, the data limitations must also be considered in the analysis of the mammal results. In the National Inland Wetland Survey, mammal taxonomic groups were surveyed only in intensive surveys, not in the general surveys. Consequently, data accumulation across regions and time periods is imbalanced. Given this data bias, future research should enhance analyses by incorporating methods to correct spatial unevenness and detection bias.

### 4.5. Implications from Cross-Taxon Drivers

The taxon-specific model analysis indicated that wetland area was a consistent positive predictor for amphibians/reptiles, birds and mammals, and NDVI also showed positive associations in these groups. To maintain and manage wetland species richness, conservation strategies tailored to each taxonomic group must be developed, considering their habitat utilization patterns. Particularly in Korea, where riverine wetlands cover over half the wetland area and factors related to wetland size and vegetation health have emerged as common critical environmental factors, protecting core river and estuary areas should be prioritized. Floodplains and riparian connectivity corridors must be managed to maintain wetland area and connectivity. Additionally, critical environmental factors that sustain populations of each taxonomic group must be periodically monitored to assess wetland health.

### 4.6. Implications for the Inland Wetland Survey: Data Structure Limitations and Future Improvements

Regarding the data from the NIWS Report, despite the abundance of survey data available for each wetland, insufficient database construction and data standardization efforts resulted in significant time and effort required during the analysis. To overcome these limitations and establish a usable national-level wetland ecological database, technical improvements and data standardization efforts are considered necessary.

First, we recommend implementing a metadata standardization module. When basic collection descriptions are missing, it is difficult to determine whether variation in SR reflects an ecological signal or differences in sampling intensity and design. Heterogeneous units, definitions, and preprocessing across reports also necessitate manual harmonization during integration, increasing the risk of error. A standardized metadata template with enforced code lists and automatic capture of core fields (e.g., survey effort, date/time, georeferenced location, investigator, and method) would mitigate these problems.

Second, a multiformat data delivery system should be established to enhance usability. All survey results should be released as narrative reports and in machine-readable format (e.g., CSV, Shapefile, and GeoJSON) along with an open API to ensure public access. Providing open-access data increases reusability and interoperability, substantially improving researchers’ abilities to discover, integrate, and analyze the data.

Third, a dataset versioning and citation system should be established to track annual updates with clear version identifiers, preventing duplication and omissions, and securing a foundation for long-term time-series analyses. Wetland recordkeeping must be standardized so that multi-year data for each site can be integrated under a single, persistent ID.

Fourth, to enable robust longitudinal inference in future work, it is important to complement externally sourced environmental layers with site-level environmental monitoring data accumulated by the surveying agency itself, consistently referenced to the survey year (e.g., water temperature, water-quality indicators such as BOD, and hydrological metrics including water level/residence time and hydroperiod), and released with standardized metadata. Linking these wetland–year environmental records to species-richness data via a persistent wetland ID would help reduce temporal mismatch in nationwide syntheses like ours and, over time, support wetland-specific change detection and evaluation of management effectiveness.

Finally, quality assurance and control workflow with automation should be introduced to flag coordinate errors, missing fields, and outliers at the point of entry. Automating the manual validation steps in this study would dramatically reduce processing time and substantially improve the reliability and reproducibility of the data. Developing and managing national-scale wetland data in this manner will improve its quality and accessibility, enhancing the reproducibility and scalability of wetland ecological research in Korea.

## 5. Conclusions

This study empirically identified structural limitations in national survey data and, using taxon-specific GLM/GAM approaches, quantified biota–environment relationships to guide ecological data standardization by analyzing 134 inland wetland survey reports compiled between 2000 and 2021. Wetland area was a consistent positive predictor for amphibians/reptiles, birds and mammals, and NDVI also showed positive associations in these groups. Moreover, format heterogeneity and missing metadata constrain time-series analyses, resulting in substantial data attrition. Because the analyses draw on secondary, report-based sources, limitations remain in survey precision, unmeasured environmental covariates, and sampling frequency. Future work should integrate standardized field protocols and be complemented by site-level environmental monitoring data accumulated by the survey institutions. Combined, these results underscore the need for a national, integrated wetland ecological database that combines standardized survey protocols, robust metadata governance, multiformat data delivery, and automated quality assurance and control.

## Figures and Tables

**Table 2:** EcoBank wetland data files with formats and attribute information (assessed Sep. 2025).

Data Name	Publish Year	Survey Period	Format	Attribute Information	Description
Wetland spatial information	2025	2022	.shp	Name, survey year, area, address, type, protected area status, centroid coordinates	Inland wetland spatial data and attribute information (2704 sites)
Intensive survey on wetland protected areas	2024	2006–2022	.xlsx	KTSN ^1^, taxon, common name, scientific name, survey year, centroid coordinates, abundance	List of biotas in reports from 2006 to 2022
Intensive survey on national inland wetlands	2023	2023	.xlsx	KTSN ^1^, taxon, common name, scientific name, survey year, centroid coordinates, abundance	List of biotas in reports from 2023
Survey of wetland biota	2022	2016–2021	.xlsx/.shp	KTSN ^1^, taxon, common name, scientific name, survey year, centroid coordinates, abundance	List of biotas in general reports from 2016 to 2021
Wetland assessment	2021	2021	.xlsx	Wetland assessment rating, name, administrative district name	National Wetland assessment rating from 2021
Wetland assessment	2020	2020	.shp	Wetland assessment rating, name, administrative district name	Assessment rating of wetlands in the 17 administrative districts

^1^ Korea Taxonomic Serial Number.

**Table 3:** Performance summary of final models by taxonomic group.

Taxa	Final Model	Sample Size	AIC	Explained Deviance (%)	Note
Amphibians/ Reptiles	Negative binomial GAM	432	1781.65	55.5	Moran’s I mitigated
Birds	Negative binomial GAM	1183	6795.70	60.1	Moran’s I mitigated
Mammals	Gaussian GLM	72	263.51	52.4	Moran’s I not significant

Notes: GAM: generalized linear model, GLM: generalized additive model, AIC: Akaike information criterion.

**Table 4:** Summary of parameter estimates for the final models by taxonomic group. Amphibians/reptiles and birds are reported from negative binomial generalized additive models (NB-GAMs), whereas mammals are reported from a Gaussian generalized linear model (Gaussian-GLM).

Explanatory Variables	Amphibians/Reptiles (NB-GAM)	Birds (NB-GAM)	Mammals (Gaussian-GLM)
Intercept	−0.1190	+2.5410 ·	−20.6791 *
Wetland area (m^2^, log)	+0.0742 ***	+0.1353 ***	+0.7154 *
NDVI	+0.7355 *	+0.3187 *	+9.5206 *
Aspect	-	+0.0022	-
Altitude (m)	−0.0001	+0.0001	+0.0066 *
TWI	-	−0.0042	−0.2789 *
Urban cover (%)	-	−0.0041 ·	-
Agricultural cover (%)	+0.0004	+0.0003	−0.0619 *
Forest cover (%)	+0.0002	+0.0008	-
Annual temperature (°C)	-	+0.0521	0.9307
Annual precipitation (mm)	+0.0001	−0.0006	−0.0079 *
Relative humidity (%)	+0.0103	−0.0117	0.3064 *
Average wind (m/s)	−0.1310	+0.0152	−2.5748 *
BOD	−0.0060	+0.0183	0.4486
Water temperature (°C)	-	−0.0173	-
Sample size (N)	432	1183	72

Notes: BOD: biochemical oxygen demand, NDVI: Normalized Difference Vegetation Index, TWI: Topographic Wetness Index. · *p* ≤ 0.1; * *p* ≤ 0.05; *** *p* ≤ 0.001.

## Data Availability

The original dataset will be provided upon request.

## Abbreviations

The following abbreviations are used in this manuscript:

AIC	Akaike information criterion
BOD	Biochemical oxygen demand
DEM	Digital elevation model
GAM	Generalized additive model
GLM	Generalized linear model
NB	Negative binomial
NDVI	Normalized Difference Vegetation Index
NIWS	National Inland Wetland Survey
NIE	National Institute of Ecology
REML	Restricted maximum likelihood
SR	Species richness
TWI	Topographic Wetness Index

## Appendix A

**Table A1:** Annual coverage of surveyed wetlands included in this study, by taxonomic group (2000–2021).

Year	Amphibians/ Reptiles (N)	Amphibians/ Reptiles (%)	Birds (N)	Birds (%)	Mammals (N)	Mammals (%)
2000	10	2.06	25	2.08	3	2.80
2001	24	4.95	38	3.17	7	6.54
2002	22	4.54	56	4.67	4	3.74
2003	23	4.74	54	4.50	3	2.80
2004	18	3.71	54	4.50	3	2.80
2005	12	2.47	54	4.50	5	4.67
2006	21	4.33	61	5.08	5	4.67
2007	22	4.54	56	4.67	5	4.67
2008	21	4.33	47	3.92	5	4.67
2009	17	3.51	48	4.00	5	4.67
2010	7	1.44	49	4.08	3	2.80
2011	10	2.06	50	4.17	6	5.61
2012	20	4.12	59	4.92	5	4.67
2013	15	3.09	57	4.75	5	4.67
2014	12	2.47	56	4.67	5	4.67
2015	22	4.54	60	5.00	7	6.54
2016	41	8.45	53	4.42	4	3.74
2017	25	5.15	48	4.00	5	4.67
2018	28	5.77	41	3.42	5	4.67
2019	37	7.63	40	3.33	5	4.67
2020	38	7.84	60	5.00	5	4.67
2021	40	8.25	134	11.17	7	6.54
Total	485	100.00	1200	100.00	107	100.00

**Table A2:** Summary of environmental variables, reference periods, and aggregation procedure used in the analysis.

Variable	Reference Period	Aggregation	Rationale
Wetland area (log)	2020–2022	Log	Wetland sizes based on the latest boundary/area standards
Wetland type	-	Category	Type (riverine, artificial, mountainous, lacustrine)
Altitude	2020	Centroid	NASADEM DEM Global 1 arc-second (~30 m) was used; altitude at the wetland centroid was used as the representative value.
Aspect	2000	Centroid	Northness (cos(aspect)), Derived from the DEM and sampled at each wetland centroid as the representative value. Transformed from aspect to account for circularity; +1 indicates north-facing and −1 indicates south-facing slopes.
Topographic wetness index	2000	Centroid	Derived from the DEM and sampled at each wetland centroid as the representative value.
Urban cover	2020	Ratio	The proportion of built-up (developed) areas was calculated within a 1 km buffer around each wetland.
Agricultural cover	2020	Ratio	The proportion of agricultural area was calculated within a 1 km buffer around each wetland.
Forest cover	2020	Ratio	The proportion of forest area was calculated within a 1 km buffer around each wetland.
Normalized Difference Vegetation Index	2019	Mean	A Landsat-based NDVI layer (30 m) distributed by the Korea Institute of Geoscience and Mineral Resources (KIGAM) for 2019 was used as a consistent proxy of vegetation greenness. NDVI was summarized as the mean value within each wetland boundary polygon; values may be lower where open-water pixels are included.
Annual temperature	1970–2000	Centroid	WorldClim climate layers summarize long-term climate conditions from interpolated monthly station data; centroid values were used to represent each wetland.
Annual precipitation	1970–2000	Centroid	
Relative humidity	1991–2020	Centroid	Station data were averaged over multiple years and interpolated to rasters; wetland-centroid values were used as representative values.
Average wind speed	1991–2020	Centroid	
Biochemical oxygen demand	2016–2023	Mean	To represent whole-wetland water conditions, the wetland-polygon mean (zonal mean) was extracted from the IDW-interpolated water-quality surface.
Water temperature	2016–2023	Mean	

**Table A3:** Sensitivity checks comparing the main Gaussian generalized linear model (Gaussian GLM) for mammal mean species richness (SR_mean) with alternative count-family models. Panel A reports model fit Akaike information criterion (AIC) for the Gaussian model fitted to SR_mean and for Poisson, negative binomial (NB), and zero-inflated NB (ZI-NB) models fitted to an integer version of the response for sensitivity purposes. Panel B summarizes whether the direction of effects for predictors retained in the final Gaussian model is consistent under the NB alternative. N denotes complete cases used for modeling, and Zeros indicates the number of zero counts in the modeled response.

Panel A. Model sensitivity (fit comparison)							
Model	Family/link			N	Zeros		AIC
Gaussian (final model)	Gaussian/identity			72	0		267.85
Poisson	Poisson/log			72	0		267.18
Negative Binomial	Negative Binomial/log			72	0		267.18
Zero-inflated Negative Binomial	ZI-NB/log			72	0		271.18
**Panel B. Direct consistency**							
Variables		Gaussian sign	Negative Binomial sign			Same direction	
Wetland area (m^2^, log)		+	+			TRUE	
Urban cover (%)		-	-			TRUE	
Forest cover (%)		+	+			TRUE	
Annual temperature (°C)		-	-			TRUE	
Relative humidity (%)		+	+			TRUE	

**Table A4:** Moran’s I test on model residuals to assess and mitigate spatial autocorrelation across taxonomic groups.

Panel A. Moran’s I diagnostics on model residuals (k = 8), before and after spatial smoothing.										
Taxa	Model stage	Final Model	Residual type	Moran’s I		Z	*p* (randomization)	*p* (mc, nperm = 1000)		Decision
Amphibians/ Reptiles	Before	NB-GLM	Pearson	0.2258		10.294	<0.001	<0.001		Spatial AC present
	After	NB-GAM + s(Xm, Ym)	Pearson	−0.0409		−1.740	0.959	0.964		Mitigated
Birds	Before	NB-GLM	Pearson	0.2155		15.151	<0.001	<0.001		Spatial AC present
	After	NB-GAM + s(Xm, Ym)	Pearson	−0.0089		−0.556	0.711	0.687		Mitigated
Mammal	Final	Gaussian GLM	Pearson	−0.0522		−0.590	0.723	0.711		No significant
**Panel B. Sensitivity of Moran’s I to neighborhood size (k) in k-nearest-neighbor weights.**										
Taxa	Model stage		k = 6 I (p_perm)		k = 8 I (p_perm)		k = 10 I (p_perm)		Conclusion	
Amphibians/Reptiles	Before (NB-GLM)		0.2494 (0.001)		0.2258 (0.001)		0.2157 (0.001)		Always significant	
Birds	Before (NB-GLM)		0.2229 (0.001)		0.2143 (0.001)		0.2024 (0.001)		Always significant	
Mammal	Final (Gaussian GLM)		−0.0737 (0.791)		−0.0522 (0.706)		−0.0584 (0.796)		Always no significant	

Notes: NB: negative binomial, GLM: generalized linear model l, GAM: generalized additive model, AC: autocorrelation.

**Table A5:** Survey domains and parameters of the National Inland Wetland Survey (NIWS) [2,27].

Project	Category	Parameter	Description
General survey (estuarine basic surveys)	General information	Information currency	Classify wetland changes into four types
		Evaluation sheet	Habitat/vegetation quality score
		Wetland baseline factsheet	Summary of wetland type, biotic environment, socioeconomic context, assessment, and special notes
	Biota	Flora	Species list, summary of biodiversity status (dominance, diversity, evenness, richness, etc.), and field photographs
		Benthic macroinvertebrates	
		Amphibians and reptiles	
		Birds	
		Fishes	
	Vegetation	Vegetation map	Map production using geographical information services (GIS); Includes these attributes (dominant species, community, distinctive vegetation, area, and physiognomic type)
Intensive survey (protected wetland area, estuarine intensive surveys)	Topography/ geology/ sedimentation	Geographic location	Geographic location Geology/lithology distribution watershed boundary
		Geomorphological characteristics	Survey using GIS spatial analysis methods Locations of inlets/outlets; distribution of bedrock and structural geology; presence/absence of surficial deposits
		Assessment of temporal change	Desk review and informant inquiry; time-series change using historical topographic maps and aerial imagery
		Major infrastructure	Presence and density of structures (dams, weirs, and small impoundments) Nearby gauging infrastructure (water-level stations, discharge gauges, sluice gates, and pump stations)
		Sediment characteristics	Sediment thickness/depth survey
		Physical-chemical properties	Laboratory analysis of collected sediments Grain-size analysis, electrical conductivity, organic matter content
	Hydrology/ hydraulics/ water quality	Hydrometeorological survey	Uses observed meteorological records to analyze interannual change, seasonal patterns, and water balance
		Hydrologic setting	Survey soil profile composition and spatial distribution; soil physical properties (e.g., porosity and hydraulic conductivity)
		Water-quality environment	Required parameters: pH, water temperature, electrical conductivity, DO, BOD, COD, SS, turbidity, TOC, T-N, T-P, and salinity
	Biota	Vegetation	Documents all plant community types Notes critical features by major vegetation types Maps spatial distribution (current vegetation map)
		Flora	Compiles a species list covering vascular and nonvascular plants (includes abundance) Flags threatened species, invasive alien plants, critical naturalized and endemic taxa; list rare/protected/indicator species and their status
		Insects	Species list (with abundance; mark threatened, endemic, indicator taxa) Community analysis
		Benthic macroinvertebrates	Species list (with abundance; mark threatened, endemic, and indicator taxa) Community analysis and aquatic habitat condition assessment
		Amphibians and reptiles	Species list (with abundance; life-stage recorded; and mark threatened taxa)
		Fish	Occurring species (abundance; mark threatened/indicator taxa) Community analysis (composition and dominance structure) Assessment of habitat conditions and diversity
		Birds	Species list (abundance; mark threatened and alien species) Diversity-index analysis Breeding status and habitat use
		Mammals	Species list; status of threatened/focal/endemic/alien species and management recommendations
		Phytoplankton/zooplankton	Species list (mark dominant, newly recorded, and indicator taxa)
		Periphytic algae	Species list and laboratory cell count results
